# Enzyme variants in biosynthesis and biological assessment of different molecular weight hyaluronan

**DOI:** 10.1186/s13568-024-01713-4

**Published:** 2024-05-10

**Authors:** Tahereh Ebrahimi, Malihe Keramati, Farnaz Khodabakhsh, Reza Ahangari Cohan

**Affiliations:** 1 New Technologies Research Group, Department of Nanobiotechnology, Pasteur Institute of Iran, Tehran, Iran; 2 Department of Genetics and Advanced Medical Technology, Faculty of Medicine, Medical Biotechnology Research Center, AJA University of Medical Sciences, Tehran, Iran

**Keywords:** Molecular weight, Hyaluronan synthase, Hyaluronic acid, Specific activity, Polydispersity

## Abstract

In the present study, low- and high-molecular-weight hyaluronic acids (LMW-HA and HMW-HA) were synthesized in vitro by truncated *Streptococcus equisimilis* hyaluronan synthases (SeHAS). The enzyme kinetic parameters were determined for each enzyme variant. The MW, structure, dispersity, and biological activity of polymers were determined by electrophoresis, FTIR spectroscopy, carbazole, cell proliferation, and cell migration assay, respectively. The specific activities were calculated as 7.5, 6.8, 4.9, and 2.8 µg_HA_ µg_enzyme_^−1^ min^−1^ for SeHAS, HAS_123_, HAS_23_, and HAS_Intra_, respectively. The results revealed SeHAS produced a polydisperse HMW-HA (268 kDa), while HAS_123_ and HAS_23_ produced a polydisperse LMW-HA (< 30 kDa). Interestingly, HAS_Intra_ produced a low-disperse LMW-HA. Kinetics studies revealed the truncated variants displayed increased K_m_ values for two substrates when compared to the wild-type enzyme. Biological assessments indicated all LMW-HAs showed a dose-dependent proliferation activity on endothelial cells (ECs), whereas HMW-HAs exhibited an inhibitory effect. Also, LMW-HAs had the highest cell migration effect at 10 µg/mL, while at 200 µg/mL, both LMW- and HMW-HAs postponed the healing recovery rate. The study elucidated that the transmembrane domains (TMDs) of SeHAS affect the enzyme kinetics, HA-titer, HA-size, and HA-dispersity. These findings open new insight into the rational engineering of SeHAS to produce size-defined HA.

**Graphical abstract:**

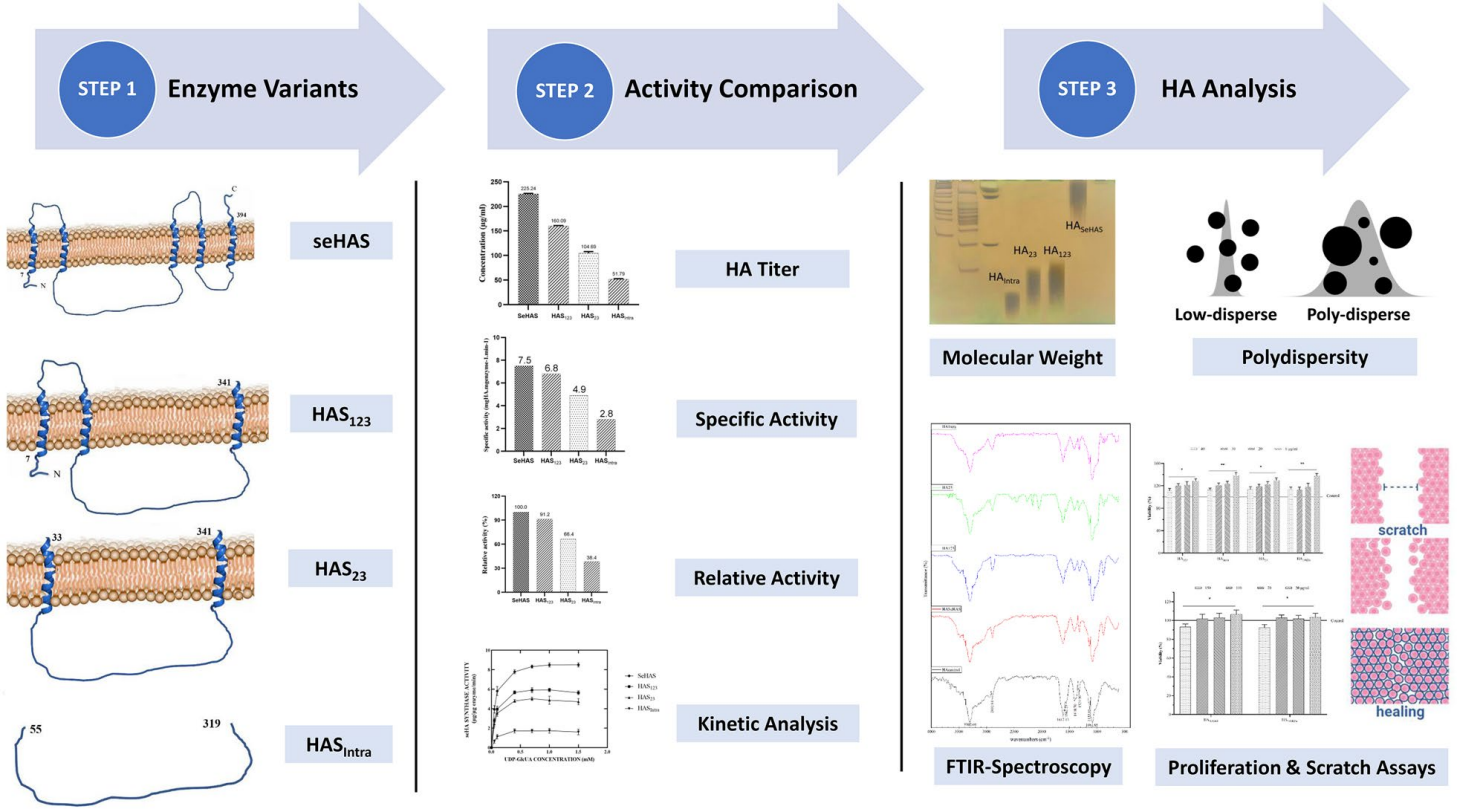

**Supplementary Information:**

The online version contains supplementary material available at 10.1186/s13568-024-01713-4.

## Introduction

Unlike the majority of biomolecules, hyaluronic acid (HA), also known as hyaluronan, is not monodispersed in molecular weight (MW). It has been established that HA with different size can have dramatically different effects on cellular signaling, functional properties, morphology and receptor binding in normal and pathological conditions (Ciccone et al. 2019; Dovedytis et al. 2020; Tavianatou et al. 2019). Depending on the molecular size, HA fragments can influence cellular behavior in a different mode of action. Physiologically, low-molecular-weight HAs (LMW-HA) (less than 200 kDa) or o-HA (Hyaluronan oligosaccharide) stimulate the proliferation and migration of endothelial cells (ECs) (angiogenic effect) (Deed et al. 1997; Sattar et al. 1994; West et al. 1985; West and Kumar 1989), whereas, high-molecular-weight HAs (HMW-HA, n-HA) have an inhibitory effect (Kumar et al. 1989; West and Kumar 1988, 1991). There have been numerous recent reports that fragments of HA have different properties compared to the intact molecule (Baggenstoss et al. 2017). This phenomenon is attributed to the different manner of interaction with cell surface receptors, especially CD44 and RHAMM (Prosdocimi and Bevilacqua 2012; Slevin et al. 2002). Both receptors can trigger signaling cascades that regulate cell functional properties, such as proliferation, migration, angiogenesis, and wound healing. Although all HA chains of different lengths are bound with the same receptor, initiation of a signal transduction cascade and diverse responses depends on HA concentration and MW (David-Raoudi et al. 2008; Mo et al. 2011). LMW-HA are able to enhance or attenuate the HA receptor-mediated signaling pathways and result in changes in gene expression, as they compete with the HMW-HA for binding to the receptors (Cowman 2017; Tavianatou et al. 2019). A deeper understanding of these mechanisms that regulate the HA fragments’ effects could contribute to future pharmacological targeting strategies (Cowman 2017).

Therefore, there is a growing interest in producing HA, emphasizing the control of polymer size and its polydispersity for answering biological questions and potentially treating diseases. HA is produced commercially based on the extraction from animal tissues and microbial fermentation using bacterial strains (Liu et al. 2011). Nevertheless, both technologies are hampered by several limitations such as avian allergens, endotoxin contamination, endogenous hyaluronidase activity, and uncontrolled degradation during extraction that limits the application of HA in the biomedical field (Boeriu et al. 2013). Recently, the main commercial methods generate natural HMW-HAs with a broad size distribution due to the intrinsic characteristics of polysaccharide biosynthesis (Sousa et al. 2009). As mentioned, to accurately interpret various biological functions of HA or synthesize better HA-based biomedical products, it is necessary to obtain a uniform size-defined or low-dispersed HA (Al-Khateeb and Prpic 2019).

Advantage of using enzymatic synthesis for HA production is the simpler downstream processing and a reduced risk of contamination. Isolated HA synthase is able to catalyze in vitro at well-defined conditions the same reaction as it catalyzes in vivo (Boeriu et al. 2013).

Numerous studies on HA production by natural bacteria have indicated that MW can be controlled by genetic (Afrasiabi et al. 2023; Jafari et al. 2022), metabolic, and process parameters (Schulte et al. 2019). On the other hand, in vitro biosynthesis of HA using recombinant hyaluronan synthases (HAS) mutants demonstrated that the control of size and polydispersity can be achieved by changing the intrinsic characteristics of the enzyme (Hascall et al. 2014; Jokela et al. 2011; Moretto et al. 2015; Yang et al. 2017). One of the intrinsic parameters that influences the size is the production capacity of the enzyme, which is determined by its amino acid sequence (Baggenstoss et al. 2017; Weigel and Baggenstoss 2012).

Although the molecular mechanism of size control of HA by HAS remains partially understood, but the control of HA size can still be achieved through manipulations of enzymatic and process parameters. In vitro enzymatic polymerization allows us to obtain both size-defined oligosaccharides and polysaccharides (Al-Khateeb and Prpic 2019; Mandawe et al. 2018).

HAS enzymes are membrane proteins with eight membrane domains (MDs) in vertebrates and 5–6 MDs in streptococcal species (Yao et al. 2021). The most streptococcal HA producers are *Streptococcus equisimilis* (SeHAS), *S. equi* subsp. *zooepidemicus*, *S. pyogenes*, *S. uberis*, *S. iniae*, and *S. parauberis*. A recent study investigated in vitro HA synthesis by streptococcal HASs with a special focus on the MW. Production of different MWs HA (0.4–1.4 MDa) by these strains could be attributed to the protein sequences of these enzymes. So, it seems that the sequence diversity of streptococcal HASs can be used for the production of molecular weight–tailored hyaluronan (Schulte et al. 2019). Hyaluronan synthases are categorized into two classes (Class I and Class II) based on their structure and function. Bacterial HASs are typically classified into Class I. Moreover, these enzymes may be considered a candidate for being engineered to regulate the HA production rate and its MW. Nonetheless, the protein engineering of class I HAS is still in its nascent stage and is mostly limited to mutation studies.

Several investigations have been performed on SeHAS, the smallest bacterial HAS (42 kDa, 417 AA), for the identification of the regions that might be involved in HA MW control (Baggenstoss et al. 2017; Yang et al. 2017). Point mutations in conserved residues corresponding to different regions of the enzyme including TMDs, central intracellular domain, and C-terminal end of the enzyme influence polymerizing activity and/or MW of the polymer. Results indicated that site-specific SeHAS mutants produced either slightly smaller or larger polymers (Baggenstoss et al. 2017). For example, the Cys mutants showed a decreased activity and produced a smaller HA $(\sim\,2.3\, \text{MDa})$ than the wild-type enzyme (3.6 MDa) (Kumari and Weigel 2005; Weigel and Baggenstoss 2012). It was reported that the substitution of two conserved polar amino acids including Lys48 (to Arg or Glu) in TMD2 and Glu327 (to Lys, Asp, or Gln) in TMD4 also influences the enzyme's activity and the MW of polymer (Kumari et al. 2006). In another study, restricted truncations and some specific mutations at the C-terminus of SeHAS showed that the synthesis activity and the MW can be independently changed (Weigel and Baggenstoss 2012; Yang et al. 2017). Indeed, it was shown that each function could be regulated and controlled by a separate enzymatic sub-mechanism. In this regard, two conserved tandem motif sequences with nine basic amino acids (B-X7-B motifs) were identified at the C-terminus of SeHAS with high affinity and specificity binding to HA (Xu et al. 2003). Any modification of these motifs can change the HA MW (Baggenstoss et al. 2017). Point mutations within the residues 414 to 417 improved the HA binding affinity and led to a larger polymer, highlighting the important role of the HA-SeHAS electrostatic interactions in the MW control (Yang et al. 2017). These findings suggested that specific mutations that alter SeHAS conformation could influence production rate and HA product MW and open new avenues for Mw-tailored HA synthesis (Agarwal et al. 2019; Mandawe et al. 2018).

Despite certain progress in the identification of C-terminal residues and motifs, there is no report on the role of each TMD on the production rate, MW, and polydispersity (Heldermon et al. 2001; Hofmann 1993). Therefore, in the current study, the effect of TMDs on the productivity and specific activity of variants, as well as the dispersity, MW, and biological activity of synthesized polymers was investigated.

## Materials and methods

### Materials, bacterial strain, plasmids, and culture conditions

Media components of Luria–Bertani (LB) broth (10 g NaCl, 5 g yeast extract, and 10 g tryptone, adjusted to 1 L with distilled H2O) and 2xYT broth (5 g NaCl, 10 g yeast extract, and 16 g tryptone, adjusted to 1 L with distilled H2O) were obtained from Ibresco (Life Science, Iran) and n-dodecyl β-d-maltoside (DDM) was purchased from Molekula GmbH (Germany). UDP-GlcUA, UDP-GlcNAc (U6751 and U4375, Sigma-Aldrich), and other reagents were supplied by Bio Basic Inc. (Canada).

### Cloning of SeHAS and variants

*Streptococcus* *equisimilis* isolate S88 group G (GGS-S88) (MW285140) was obtained from the previous study (Cohan et al. 2023). This strain was used for DNA genomic extraction. The genes of SeHAS and variants were amplified using specific primers (Additional file 1: Table S1). The genes were then cloned into pET-28a (+) (Novagen, Germany) and transformed into *E. coli* BL21 (DE3) strain (Novagen, Germany) (Additional file 1: Fig. S1). To facilitate the purification of SeHAS variants, a C-terminal fusion of 6-His residues was added to each construct. The recombinant hosts were grown on LB broth or LB agar containing 50 µg/mL kanamycin. The summarized properties of variants used in the present study were shown in Additional file 1: Table S2. The schematic membrane topology of variants is depicted in Additional file 1: Fig. S2.

### Expression and purification of variants

The cultures were induced with 1 mM IPTG and incubated for 18 h at 37 °C. The cells were centrifuged and the pellets were washed twice with phosphate-buffered saline (PBS). For purification of HAS_Intra_, a hybrid method was used. First, the pellet was resuspended in 12 mL lysis buffer (8 M Urea, 0.5 M NaCl, 72 mM K_2_HPO_4_, 17 mM KH_2_PO_4_, pH 7.5) and incubated on ice for 40 min. The sonicated cell (three times, 30s at 20 W) was centrifuged at 12,000 g for 20 min. The supernatant was transferred to Ni–NTA resin (Sigma Inc., USA) equilibrated with lysis buffer. The mixture was incubated for 90 min at 4 °C with constant mixing. After incubation, the mixture was then washed two times with wash buffer I (lysis buffer containing 3 M Urea, pH 7.5) and wash buffer II (lysis buffer with 100 M NaCl, pH 7.5), respectively. The protein was eluted with elution buffer (72 mM K_2_HPO_4_, 17 mM KH_2_PO_4_, 300 M NaCl, 250 mM Imidazole, pH 7.5). Total protein concentration for HAS_Intra_ was determined by Bradford assay using bovine serum albumin (BSA) as standard. The expression of HAS_Intra_ was confirmed by 12% SDS-PAGE and western blot (anti-His antibody, Sigma, USA) according to standard procedures (Sambrook et al. 1989). In our previous study, we used an ionic detergent, sodium dodecyl sulfate (SDS), for the solubilization of membrane proteins (Cohan et al. 2023). However, SDS has a deleterious effect on the protein conformation that leads to the denaturation of protein. Therefore, for membrane proteins (SeHAS, HAS_123_, and HAS_23_), we examined the effects of non-ionic detergents such as DDM, Triton X-100, and Tween 20. The concentration of purified variants was carried out using Bicinchoninic acid (BCA) protein assay kit (Pierce) using bovine serum albumin as a standard (Smith et al. 1985). Unlike Bradford method, BCA assay is compatible with a broad range of detergents at high concentrations (Orwick-Rydmark et al. 2016).

### Determination of Michaelis–Menten constants

The Michaelis–Menten (K_m_) values for the two substrates were determined using detergent-solubilized purified SeHAS variants. The enzyme activities were determined in 100 µl of 25 mM sodium and potassium phosphate (pH 7.0) containing 50 mM NaCl, 20 mM MgCl_2_, 1.0 mM dithiothreitol, 1.0 mM EDTA, 2 M glycerol, 1 mM n-dodecyl-β-d-maltoside, 1 mM UDP-GlcUA, and 1.0 mM UDP-GlcNAc. Based on previous experiment, 1 mM of each substrate was found to be optimum for biosynthetic reaction. Therefore, for the determination of Km value, the excess of each substrate was considered at 1.5 mM concentration. The K_m_ values for both substrates (Km_UDP-GlcUA_ and Km_UDP-GlcNAc_) were determined in duplicate by varying one substrate while keeping the other constant using either Lineweaver–Burk or Hill analysis (Hill 1910). The purified enzyme variants (0.1 µM) were added to initiate the reaction and the mixture was gently agitated at 30 °C for 1 h. The reactions were terminated by the addition of sodium dodecyl sulfate (2% w/v) (Tlapak-Simmons et al. 2004). The kinetic parameters (Km and Kcat) were determined using different substrate concentrations (ranging from 0.05 to 1.5 mM) under same conditions by GraphPad Prism 6 using Michaelis–Menten kinetic equations.

### HA production and purification

HA production by SeHAS and its variants was performed in a reaction (100 µL) containing 25 mM sodium and potassium phosphate, 50 mM NaCl, 20 mM MgCl_2_, 1.0 mM DTT, 1.0 mM EDTA, 2M glycerol, 1mM DDM, 1 mM UDP-GlcUA, and 1.0 mM UDP-GlcNAc, adjusted to pH 7.0. To initiate the reaction, 0.1 µmol of each variant was added and the mixture was gently mixed at 30 °C. After 60 min incubation, the reactions were terminated by the addition of SDS at a final concentration of 2% w/v (Tlapak-Simmons et al. 2004). For HA purification, 0.15 M NaCl solution was added to the reaction mixtures. Then, the mixtures were cooled to 4 °C for 60 min to form the HA salt. The HA salt was precipitated by adding three volumes of ethanol followed by incubation at 4 °C for 24 h. The resulting HA salt was collected by centrifugation (12,000 g, 20 min at 4 °C) and resuspended in distilled water (Cavalcanti et al. 2019; Rodriguez-Marquez et al. 2022). The protein impurities were determined using a UV/Vis spectrophotometer (Thermo Fisher, USA). To investigate the effect of the purification process on HA Mw, the purification step was also performed on a control HA with a MW of 760 kDa (Bloomage Corporation).

### HA quantification

HA titer was determined by carbazole assay with some modifications (Bitter 1962; Cesaretti et al. 2003). Briefly, a serial dilution of standard (0–500 µg/mL, d-glucuronic acid, Sigma) and sample solutions (50 µl) was prepared in a 96-well microplate. Then, 200 µL sodium tetraborate solution (0.025 M in saturated sulfuric acid) was added to the wells and mixed gently. The microplates were heated for 20 min at 80 °C. After cooling at room temperature, 50 µL carbazole (0.125% in absolute ethanol) was added to each well and mixed well. The microplate was read in a microplate reader (Metertech, Taiwan) at a wavelength of 550 nm after heating at 80 °C for 20 min in an oven and cooling at room temperature for 15 min.

### FIIR spectroscopy

Previous studies showed that the identification and structural analysis of HA products can be assessed using FTIR spectroscopy (Afrasiabi et al. 2023; Amjad Zanjani et al. 2022; Chahuki et al. 2019; Gilli et al. 1994; Karami et al. 2021; Song et al. 2023). For this purpose, the purified polymers samples and control were analyzed by a FTIR apparatus (Thermo, USA) in a wavenumber range of 4000–400 cm^−1^ under the same operational conditions.

### Polydispersity and MW determination

The polydispersity of HA samples and the control samples (HA_10kDa_ and HA_760kDa_) (Bloomage Corporation) was evaluated by dynamic light scattering (DLS) technique (Malvern, Nano-Zs) (Dodero et al. 2018). The HA MWs were determined by polyacrylamide gel electrophoresis (PAGE) with a combined Alcian blue and silver staining reported by Min and Cowman with some modifications (Min and Cowman 1986). The mini-slab gels of 8 × 9 × 0.1 cm containing 15% acrylamide, 0.5% N, Nˊ-methylene bis-acrylamide in 0.1 M Tris–borate-1 mM Na_2_EDTA, pH 8.3 (Tris/borate/EDTA) were used. The purified samples were dissolved in water and mixed with a one-fifth volume of 2 M sucrose in Tris/borate/EDTA buffer and loaded on the gel. Bromophenol blue (0.005% in Tris/borate/EDTA buffer containing 0.3 M sucrose) was used as a tracking dye and LMW-HAs (Echelon Biosciences Inc., HYA-LOLAD) marker was used as a ladder. The gels were run at 170 V for 30 min, then at 250 V for 10 min, and finally at 200 V $\text{for}\,\sim\,40\,\text{min}$ until the tracking dye reached within 1 cm of the gel bottom. The electrophoretic process was carried out at 4 °C (Ikegami-Kawai and Takahashi 2002). Immediately after electrophoresis, the samples were fixed in the gel by soaking the gel in 0.5% w/v Alcian blue in 2% w/v acetic acid, for 30 min far from light. After destaining in water for 30 min, the gel was subjected to silver staining, beginning from the oxidation step, using the Bio-Rad silver stain kit according to the manufacturer’s protocol (Min and Cowman 1986). In brief, the gel was soaked for 10 min in a 200 mL solution of 0.0034 M potassium dichromate and 0.0032 N nitric acid. It was washed two times for 20 min in 200 mL of deionized water and placed in 100 ml of 0.012 M silver nitrate for 20 min. This step was followed by rapid rinsing with 100 mL of the image developer solution, which contains 0.28 M sodium carbonate and 0.5 ml of commercial formalin per liter. The gels were gently shaken in this solution until the HA bands appeared. The development was stopped by discarding the developer and addition of 100 ml 5% w/v acetic acid. The gels were washed twice with 200 ml of water before storage and sealed in plastic bags (Merril et al. 1981).

### Proliferation assay

MTT assay was used to investigate the effect HA products on cell proliferation. Adherent human umbilical vein endothelial cells (HUVEC, NCBI code: C554) were seeded at a density of 1.0 × 10^4^ cells/well in the specific medium containing 10% fetal bovine serum (FBS, Gibco U.S.A). After the cell growth, the medium was replaced with HA products, LMW-HA (HA_10kDa_), and n-HA (HA_760kDa_) at different concentrations (180 μL preparations in medium supplemented with 10% FBS). The plates were then incubated for 48 and 72 h. After that, 20 μL methylthiazolyldiphenyl-tetrazolium bromide (MTT, T0793 Bio Basic Canada) reagent was added to each well, and the plates were incubated for 4.0 h at 37 °C. The supernatants were discarded and isopropanol was added to each well. The microplates were shaken in an oscillator at room temperature for 15 min and the absorbance was measured using an Epoch microplate spectrophotometer (BioTek, USA) at 570 nm. The experiment was repeated three times and the results were expressed as the percentage of viability when compared to untreated cells (Negative control is considered as 100% of viability).

#### Cell migration assay

HUVEC were plated at a density of 1.0 × 10^5^ cells/well in a specific medium containing 10% FBS in 12 wells plates. After reaching a cell density of 1.0 × 10^9^ per well, the medium was removed and the cells were rinsed with phosphate-buffered saline (PBS, pH 7.0). Then, a sterile pipette tip was held vertically to scratch a line in each well. The detached cells were removed by washing with PBS. After that, the HA products at final concentrations of 10 and 200 µg/mL were added to the wells. The controls were 180 µL culture medium without HA, n-HA (HA_760kDa_), and LMW-HA (HA_10kDa_). The scratch closure was monitored and imaged at 5-time points for 24 h using a BEL INV-2 microscope (BEL Engineering, Italy) (Wang et al. 2016). The results were expressed as the percentage of wound closure with respect to starting time point, quantified using an image-analysis program (ImageJ, version v1.54d) (Suarez-Arnedo et al. 2020).

#### Statistical analysis

Statistical analysis was performed using Student’s t-test to compare two groups or one-way ANOVA in the case of three or more groups with GraphPad Prism. All data are presented as mean ± SD from three independent experiments. A *p*-value less than 0.05 was considered a statistically significant difference between the samples.

## Results

### Purification and activity of variants

HAS_Intra_ was expressed and purified as a ∼30 kDa non-membrane bond protein (Additional file 1: Fig. S3). The purification of membrane proteins is challenging due to their low solubility in conventional detergents. The efficacy of detergent usually depends on the membrane protein type and the purification conditions. For this reason, in the current study, different nonionic detergents including DDM, Triton X-100, and Tween 20 were applied to compare the extent of protein solubilization. In our previous study, we purified the membrane variants using SDS as a surfactant. However, as our data revealed, the loss of protein, as well as the reduction in activity, was remarkable. Data illustrated that all detergents could solubilize the bacterial membrane. However, DDM led to more solubilization (Additional file 1: Table S3). Nonetheless, more solubilization by a detergent may not necessarily means maintaining protein structure and activity. Therefore, the activity of variants was quantitatively evaluated by measuring the amount of HA in vitro synthesis. The concentration of HA in individual reactions, as well as the specific enzyme activity of variants was calculated. Data revealed that the variants purified by Triton X-100 and Tween 20 have a remarkable reduction in activity when compared to that of the DDM-purified (Additional file 1: Table S3). In DDM-based purification, although all variants kept the biosynthesis capability, however, they showed lower specific activity rather than the wild-type (Table 1). HA titer was statistically different among the variants, so that SeHAS and HAS_Intra_ had the highest and lowest HA titer, respectively (Additional file 1: Fig. S4). Determination of protein impurities in HA samples demonstrated that all are in the range of acceptance limit according to *European Pharmacopoeia 10*. The purification steps did not have deleterious effects on HA Mw when tested by the control.

**Table 1:** HA titer and specific activity of recombinant variants after purification with DDM

Enzyme	Productivity (μg_HA_/nmol_enzyme_)	Specific activity (µg_HA_ µg_enzyme_^−1^ min^−1^)	Relative activity (%)
SeHAS	25.71	7.5	100.0
HAS_123_	23.44	6.8	91.2
HAS_23_	17.08	4.9	66.4
HAS_Intra_	9.86	2.8	38.4

### Enzyme kinetics

**Fig. 1:**
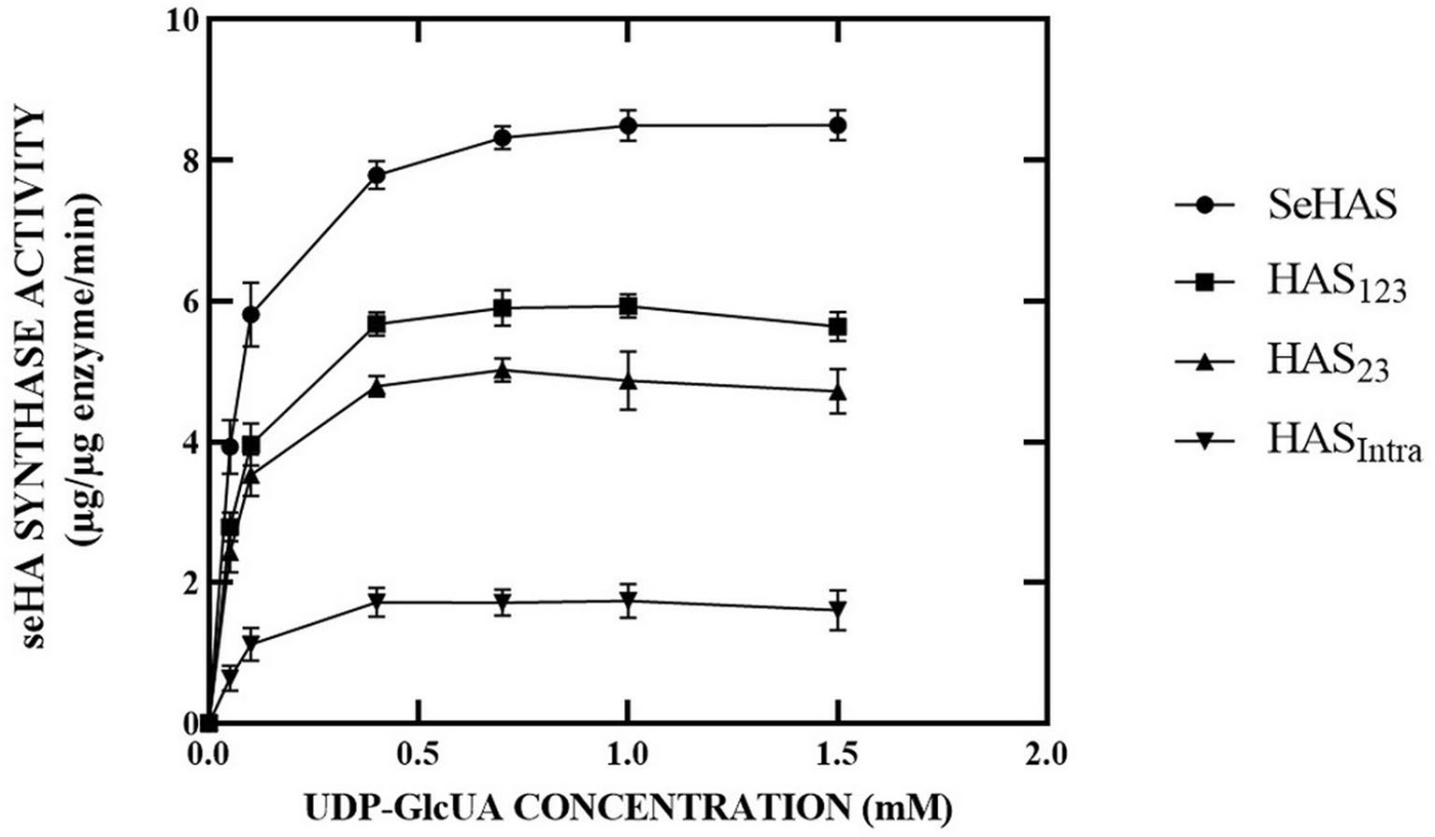
Effect of UDP-GlcUA concentration on the activity of SeHAS and truncated forms. The K_m_ values for UDP-GlcUA were determined by varying the concentration of UDP-GlcUA from 0.05 to 1.5 mM while keeping the other at 1.5 mM. As depicted, all saturation profiles are hyperbolic

**Fig. 2:**
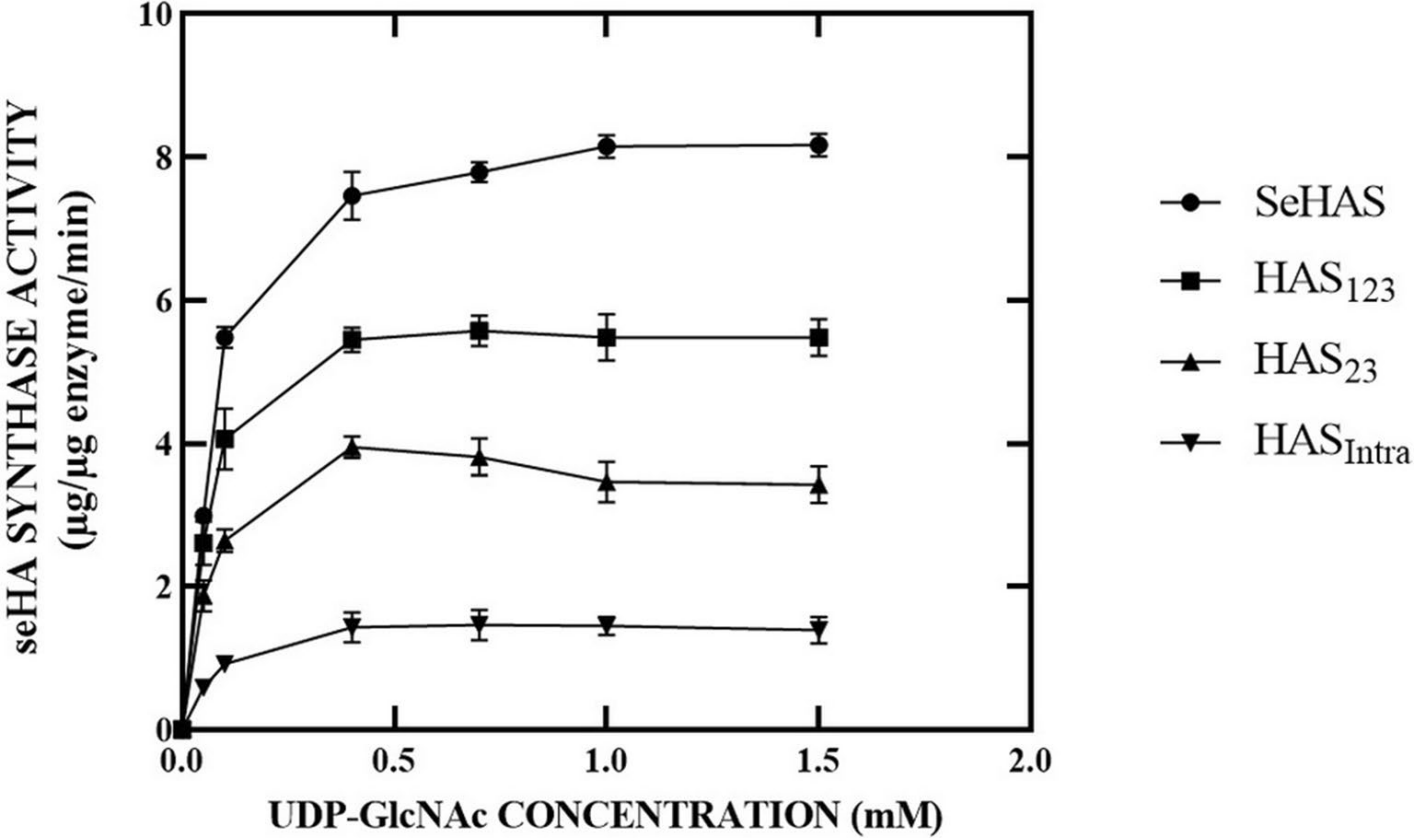
Effect of UDP-GlcNAc concentration on the activity of SeHAS and truncated forms. The K_m_ values for UDP-GlcNAc were determined by varying the concentration of UDP-GlcNAc from 0.05 to 1.5 mM while keeping the other at 1.5 mM. As depicted, all saturation profiles are hyperbolic

The kinetic behaviors of SeHAS variants are shown in Figs. 1 and 2. As noted in previous studies, SeHAS appeared to be more catalytically active than the truncated forms (Cohan et al. 2023; Tlapak-Simmons et al. 2004). The substrate concentration significantly alters the activity of variants (*p*-value < 0.0001). The activity of SeHAS and truncated forms at different UDP-GlcUA concentrations were 8.5, 5.6, 4.7, and 1.6 µg_HA_ µg_enzyme_^−1^ min^−1^ for SeHAS, HAS_123_, HAS_23_, and HAS_Intra_, respectively. Similarly, the activity of SeHAS and truncated forms at different UDP-GlcNAc concentrations were 8.2, 5.5, 3.4, and 1.4 µg_HA_ µg_enzyme_^−1^ min^−1^ for SeHAS, HAS_123_, HAS_23_, and HAS_Intra_, respectively. The K_m_ values for both substrates differed slightly at the saturated concentration (Table 2). Statistical analysis indicated significant differences in the Michaelis constants of Km_UDP-GlcUA_ (*p*-value < 0.0001), suggesting that the variance in the binding affinity of the two UDP-sugars controls the molecular weight of hyaluronan. The V_max_ profiles for all enzymes were hyperbolic, while the Lineweaver–Burk plots were linear (Additional file 1: Fig. S5).

**Table 2:** The Michaelis–Menten constants obtained for SeHAS and the truncated forms

Enzyme	Km_UDP-GlcUA_ (mM)^a^	Km_UDP-GlcNAc_ (mM)^a^	V_max_ (nmol/μg/h)^b^	K_cat_ (V_max_/[Et]^d^) (Min^−1^)^c^	Kcat/Km_UDP-GlcUA_	Kcat/Km_UDP-GlcNAc_
SeHAS	0.050 ± 0.00	0.055 ± 0.01	1.00 ± 0.06	10.01	200.20	182.00
HAS_123_	0.063 ± 0.00	0.058 ± 0.01	23.83 ± 2.09	238.30	3782.54	4332.73
HAS_23_	0.065 ± 0.00	0.060 ± 0.00	23.59 ± 1.10	235.90	3629.23	4289.09
HAS_Intra_	0.078 ± 0.01	0.070 ± 0.01	17.56 ± 1.12	175.60	2251.32	3192.73

^a^Concentration of the substrate at which half of the active sites of the enzyme are occupied by the substrate

^b^The maximal velocity values for the purified HASs

^c^Catalytic activity of the purified HASs

^d^Et 0.1 µM

### FTIR spectroscopy

**Fig. 3:**
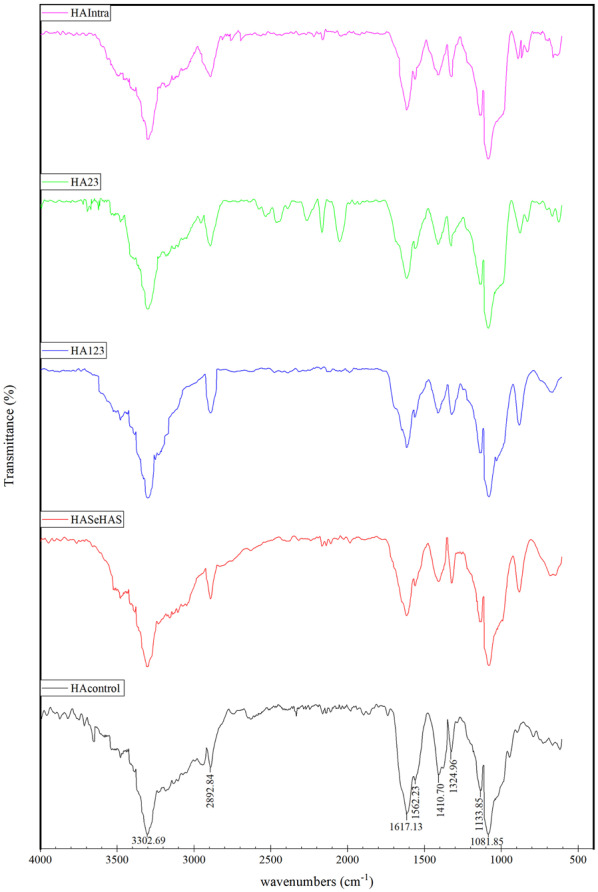
FTIR spectroscopy of purified HAs from SeHAS, HAS_123_, HAS_23_, and HAS_Intra_ along with control

The structural identity of synthesized polymers was determined by FTIR and compared to that of the control. The spectra analysis elucidated that there is no obvious difference between the spectra of HA control and purified HAs (Fig. 3). A strong absorption peak was observed at 3302 cm^−1^, which indicates OH and NH bonds. The absorption peak at 2893 cm^−1^ was related to CH symmetrical and CH2 asymmetrical stretching. The peaks at positions 1617 cm^−1^, 1562 cm^−1^, and 1324 cm^−1^ can be for amides I, II, and III. The absorption peaks at 1081 cm^−1^ and 1133 cm^−1^ are typical for carbohydrates and the peak at 1410 cm^−1^ is assigned to symmetric C–O stretching vibrations (Chen et al. 2019; Gilli et al. 1994).

### MW and dispersity of polymers

**Fig. 4:**
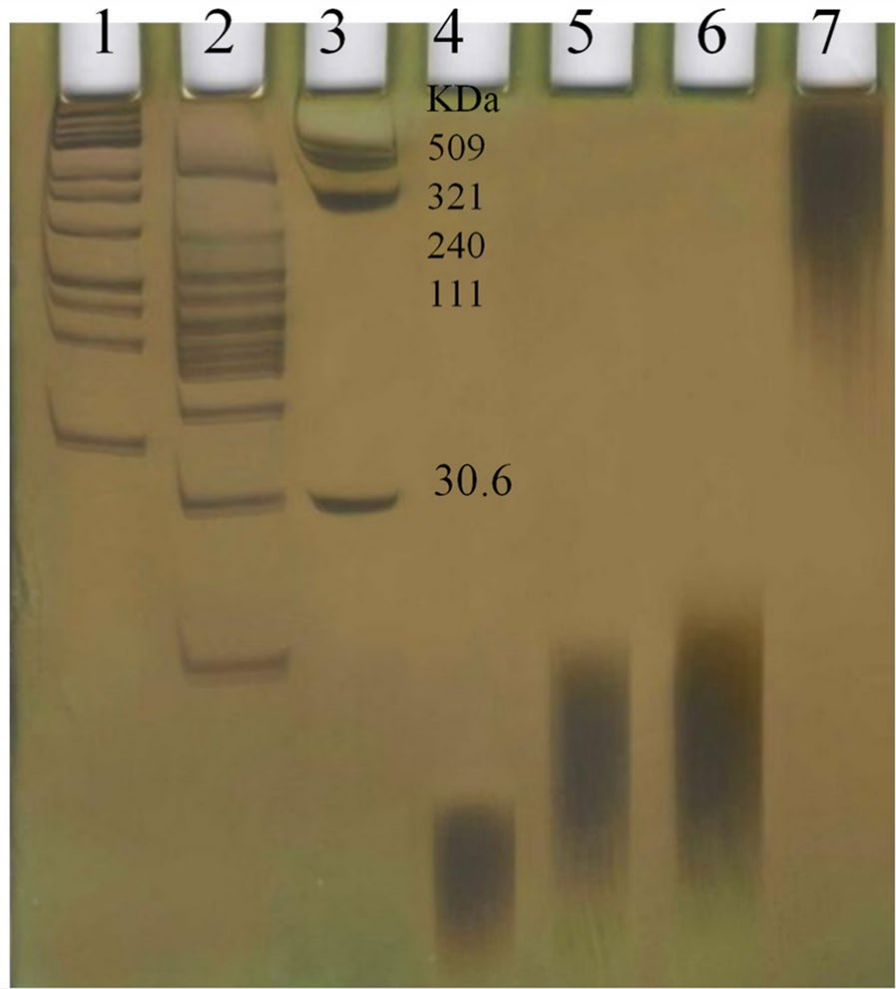
MW determination of HA products [Lane 1: DNA ladder 1 kb, Lane 2: DNA ladder 100 bp, Lane 3: HA ladder (a range from 509 to 30.6 KDa), Lane 4: HA produced by HAS_Intra_, Lane 5: HA produced by HAS_23_, Lane 6: HA produced by HAS_123_, and lane 7: HA produced by SeHAS]

The MW of HAs was determined by PAGE and Alcian blue/silver staining. SeHAS produced a HMW polymer (> 270 kDa), meanwhile HAS_123_, HAS_23_, and HAS_Intra_ produced LMW ones (< 30 KDa) (Fig. 4). The dispersity of HA polymers is summarized in Table 3. DLS experiments showed the presence of a low-disperse polymer (PDI < 0.3) for HAS_Intra_ product (HA_Intra_) and a polydisperse HA (PDI range: 0.5–1.0) for other products.

**Table 3:** Dispersity and MW of produced HAs in this study

HA sample	MW (kDa)	Size distribution
HA_SeHAS_	268	Polydisperse
HA_123_	< 30	Polydisperse
HA_23_	< 30	Polydisperse
HA_Intra_	< 30	Low-disperse
HA_10kDa_ (LMW-HA control)	10	Low-disperse
HA_760kDa_ (HMW-HA control)	760	Polydisperse

### Endothelial cell proliferation assay

**Fig. 5:**
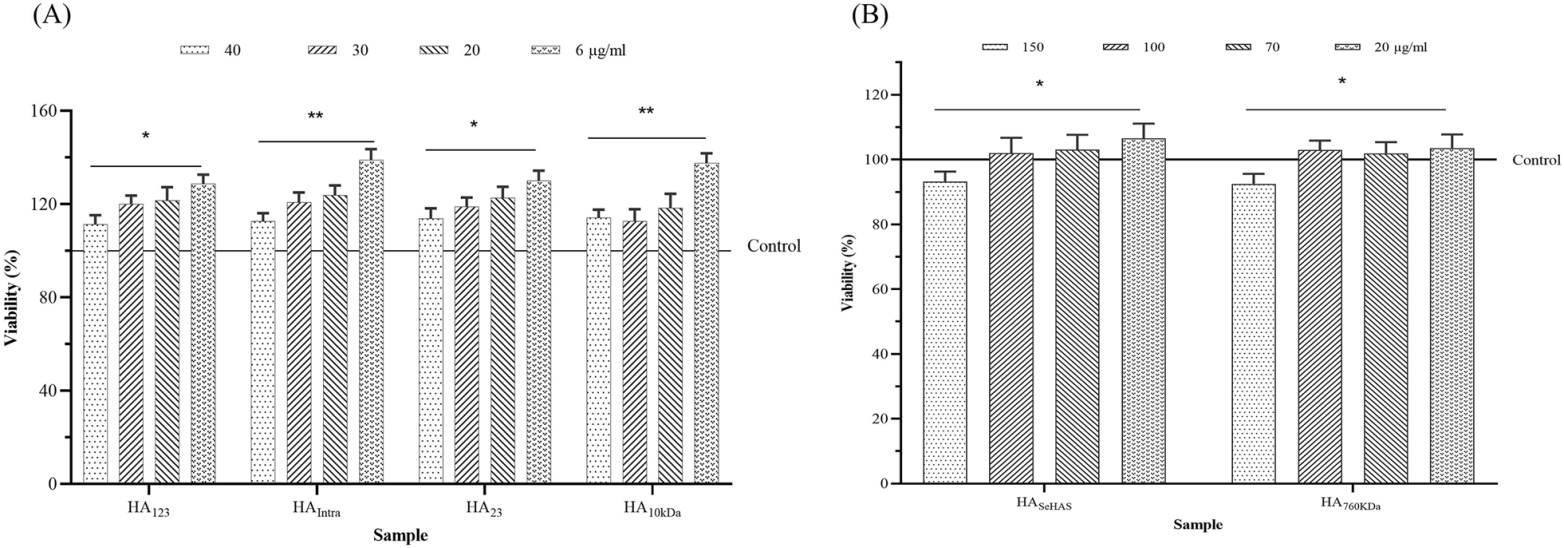
Cell proliferation assay of synthesized HAs. ECs were exposed to LMW- and HMW-HAs at concentrations of 6–40 µg/mL (**A**) and 20–150 µg/mL (**B**), respectively. Data were expressed as mean ± SD from three independent experiments. Statistical comparisons were performed between the lowest concentration relative to the highest concentration (*p-value < 0.05; **p-value < 0.01)

HUVECs were stimulated by various concentrations of the synthesized polymers. EC proliferation was negligible after 48 h for all LMW-HAs (data not shown). While, a reverse dose-dependent cell proliferation was occurred after 72 h by LMW-HAs (Fig. 5A). The highest proliferation was for HA_Intra_ and HA_10kDa_ (LMW-HA control) at concentration of 6 µg/mL. In contrast, HA_SeHAS_ and HA_760kDa_ (HMW-HA control) showed an inhibitory effect at 150 µg/mL, while lower concentrations (100, 70, and 20 µg/mL) did not affect the EC proliferation (Fig. 5B).

### In vitro cell migration assay

**Fig. 6:**
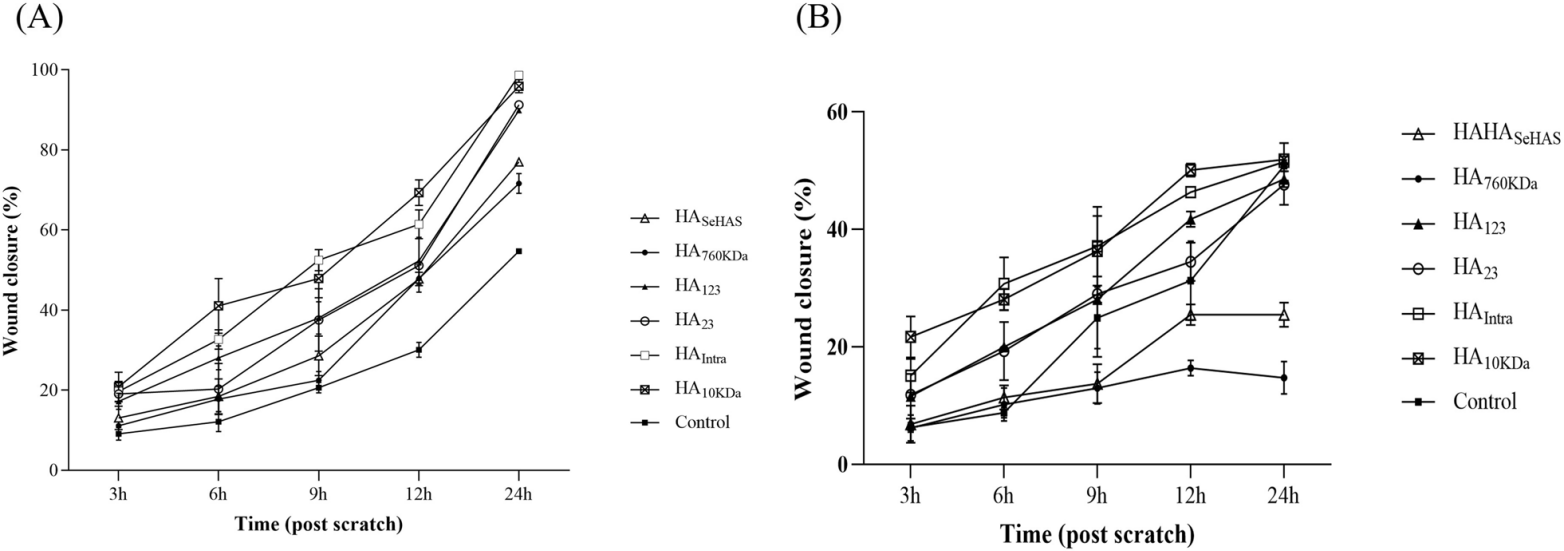
Cell migration assay. ECs were exposed to the produced HAs at concentrations of **A** 10 µg/mL and **B** 200 µg/mL. The graph reports the percentage of wound closure relative to time zero. Data were expressed as mean ± SD from three independent experiments

The scratched cells were treated with HA samples at two doses (10 μg/mL and 200 μg/mL) and the lesion closing rates were monitored for 24 h. The results showed that the scratched area began to close at 24 h after the addition of exogenous 10 μg/mL LMW-HAs/ HA_10kDa_, which was in parallel with the EC proliferation test (Fig. 6A). Whereas in other groups (HA_760kDa_ and culture medium), the lesion did not fully close after 24 h (Fig. 6B). Comparing the two doses showed that the lesion healed faster at the lower dose in both LMW-HAs and HMW-HAs groups. The cell imaging demonstrates that the wound undergoes negligible proliferation after treatment with a high concentration of HAs (independent of size) (Additional file 1: Fig. S6).

### Discussion

The MW of HA dictates its biological function (Ebrahimi et al. 2022; Garantziotis and Savani 2019). Therefore, there is great interest in understanding the mechanisms and factors influencing the size of HA. Moreover, the cell signaling capabilities of LMW-HA have encouraged producers to synthesize the lower mass of the polymer (Simpson et al. 2015). In addition to the generation of smaller HA by degradation of the long-chain polymers, HAS itself could be regulated directly to synthesize smaller HA (e.g. 40–100 kDa) (Baggenstoss et al. 2017; Hascall et al. 2014; Jokela et al. 2011; Moretto et al. 2015; Yang et al. 2017). Despite basic knowledge about HAS, however, major questions regarding the mechanism of synthesis and control of size have still remained unanswered. In total, HAS has intrinsic properties that influence both the rate and size of HA. Thus, these two functions could be regulated and controlled by separate sub-mechanisms (Baggenstoss et al. 2017). Sequence alterations as an intrinsic factor play a role in Mw variations so, the enzyme could be regulated to directly synthesize LMW-HA (Schulte et al. 2019). The hypothesis for the present study is that TMD deletions probably by altering the enzyme structure could control the HA size.

In the current study, we truncated the SeHAS enzyme to make a more detailed investigation of the function of the transmembrane domains. Extended deletion methodology was used to investigate the effects of TMDs on the preparation of LMW-HA at in vitro conditions.

The pioneering work of Dorfman and co-workers in the 1950s and 1960s showed that the streptococcal HAS is located in the cell membrane, requires Mg^2+^ ions, and uses two sugar nucleotides (UDP-GlcUA and UDP-GlcNAc) to polymerize a HA chain (Markovitz et al. 1959; Stoolmiller and Dorfman 1969). All of the known enzymes catalyze reactions that use one or two (or, rarely, three) substrates and produce one or two products. However, hyaluronan synthases (HASs) are different from the characterized enzymes. HASs possess dual glycosyltransferase activities within a single protein, (β1, 4-GlcNAc transferase and β1, 3-GlcUA transferase), and the hyaluronic acid (HA) product sequentially serves as the acceptor for each subsequent sugar addition. Although it seems straightforward, the enzyme must possess at least six (and probably seven) different functions to perform the overall reaction for the synthesis of one disaccharide unit. HAS enzymes possess an additional distinctive feature, as the structural similarity between the two sugar nucleotides affords each the potential to engage in competitive binding for the appropriate UDP-sugar-binding site on the enzyme. Although the HASs do not misincorporate other sugar nucleotides into the growing HA chain, UDP-GlcUA can interact with the UDP-GlcNAc binding site (Tlapak-Simmons et al. 1999b). Initial experiments have indicated that with the two normal substrates, a significant disparity in the concentration of one UDP-sugar compared to the other leads to variations in the rate of hyaluronic acid (HA) synthesis (Tlapak-Simmons et al. 1999b). There are no previous reports of this cross-talk phenomenon affecting HA biosynthesis between SeHAS and their truncation variants. The observation of this kinetic behavior reflects the advantage of studying purified SeHAS truncation forms and catalytic efficiency comparison between enzyme forms. Here, for the first time, we have characterized the kinetic behavior of purified SeHAS truncated forms. The catalytic region for SeHAS was reported in 264 AA from positions 55 to 319. In the present study, the SeHAS variants were truncated from the N-terminal and C-terminal extremity based on the folding structure. Studies have shown that the intracellular domain has an important role in polysaccharide synthesis. Polysaccharide-synthesizing capability of this domain could be retained depending on the truncation size. The result showed the enzymes displayed a decreasing activity depending on the size of truncation. Our data were similar to the previous studies in that the manipulated enzymes could still produce HA polymer, but differ in the amount of synthesized HA due to differences in the enzyme activity.

Indeed, HAS enzymes are highly lipid-dependent and are most effectively stimulated by cardiolipin. Tlapak-Simmons conducted a study on the influence of cardiolipin on the kinetics of Km_UDP-GlcNAc_ in the purified SeHAS. In the absence of cardiolipin, the purified SeHAS was active and had responses to increasing substrate concentration (Tlapak-Simmons et al. 1999a). A significant enhancement in the synthase activity observed in the presence of cardiolipin may be attributed to a cardiolipin-dependent influence on the enzyme, resulting in alterations in the Km values (four–ten-fold increase) for the sugar nucleotide substrates. They also reported the Km_UDPGlcUA_ and Km_UDP-GlcNAc_ values respectively as 274 µM and 251 µM for detergent-solubilized SeHAS in the presence of 2 mM bovine cardiolipin. They found a hyperbolic behavior for purified SeHAS in response to increasing substrate concentration as shown in our study. However, the reported K_m_ values are much higher than our obtained values. Therefore, the addition of bovine cardiolipin can be explained by such an increase in the specific activity of purified SeHAS. This conclusion is supported by the results of this study showing that cardiolipin-depleted SeHAS, still has enzyme activity. Furthermore, the results demonstrated that the n-dodecyl-d-maltoside-solubilized purified variants were active. This is an important finding because many studies have reported that HAS activity is irreversibly lost upon solubilization of the protein in a wide variety of nonionic detergents (Tlapak-Simmons et al. 1999b).

Interestingly, two substantial differences were observed between SeHAS and the truncated forms. First, the SeHAS is intrinsically more active than the truncated ones. This was apparent in the current and the earlier studies (Cohan et al. 2023; Keramati et al. 2022) that were conducted with both membrane-bond and purified enzymes. With the addition of UDP-sugars, truncated enzymes underwent a glycosylation reaction and transferred the substrate to the acceptor effectively. Second, the truncated variants displayed increased K_m_ values for two substrates when compared to the wild-type enzyme. SeHAS without any deletion showed lower Km values, whereas HAS_Intra_ with completely deleted transmembrane domains showed a significantly higher Km value. Kinetic analysis showed that both Km and Kcat of HAS_Intra_ were affected significantly. This is an effective way to make use of HAS_Intra_ as the shortest enzyme form for HA biosynthesis. The intracellular region has a key role in maintaining SeHAS activity. These results indicate that the presence of TMDs is essential for substrate-binding affinity and deletion of each domain increases the K_m_ value. The kinetic analysis indicated that the low affinity for substrate in the truncated forms regulates the hyaluronan molecular weight and will have a significant effect on the polydispersity of the products (Kooy et al. 2014). Furthermore, these results offer several possible strategies to decrease the polydispersity of the products including metabolic engineering to change the substrate concentration in HA-producing microorganisms and/or single mutation studies to regulate the binding affinity of the substrate within the HAS enzyme.

Multiple groups have created streptococcal HAS mutants to identify property-influencing sites such as activity and HA size (Baggenstoss et al. 2017). Mutational studies involving point-mutation within TMDs and the C-terminus of SeHAS showed different effects on HA synthesis and/or product size (Kumari et al. 2006, 2002). It was demonstrated disrupting the interaction between TMD_2_ and TMD_4_ caused the synthesis of smaller polymer (0.6–3.2 MDa versus 3.6 MDa) (Kumari et al. 2006). Some studies examined site-specific mutations in the C-terminus of SeHAS to evaluate the role of this region in the activity and MW control (Heldermon et al. 2001). Mutation’s study showed that polymerizing activity can decouple from the size control. In this regard, a tandem motif region (K^398^–X_7_–R^406^–X_7_–K^414^ motif) at the C-terminus of the enzyme was deciphered. They find almost R406 mutants synthesize a larger polymer and the variants show specific activities from 70 to 177% of the native enzyme. While the K398 mutants were associated with a remarkable reduction in the specific activity (14–64% of wild type) that led to a smaller size of polymer (≤ 250–480 kDa) (Baggenstoss et al. 2017). In a similar study, mutations in the last nine residues at the C-terminal of SeHAS (409–417) showed a decrease in the HA titer (16.8% of WT) and MW (< 790 kDa), emphasizing the importance of the C-terminus region for HAS activity and size regulation (Yang et al. 2017). Moreover, this study demonstrates how R406-R413 residues constituted an HA-binding pattern that stabilizes the HA-SeHAS complex and helps the enzyme to regulate the production rate and molecular size of the polymer. They increased the HA product size via site-directed mutation at the C-terminal (residues 414 to 417) based on the hypothesis that higher binding affinity between the SeHAS C-terminus and HA would lead to a larger HA size. A three-fold increase in the size (HA MW = 1270 kDa) due to enhanced binding affinity in the K414R variant suggested that residues 414–417 are involved in polymer retention to make a longer chain (Yang et al. 2017). In our previous study, we focused on the role of two C-terminus TMDs (TMD_4_ and TMD_5_) of SeHAS in the recombinant *Bacillus subtilis* strain and find out that the deletion of these transmembranes did not affect the MW of polymer (Amjad Zanjani et al. 2022).

It was found that C-terminal end deletion has no effect on HA synthesis activity, but it will improve the stability of the enzyme-HA protein complex and lead to the production of HMW-HAs. However, these studies have been limited to the C-terminus region of SeHAS. Therefore, in the current study, we employed extended TMD deletions to clarify the role of other domains in the activity of enzyme and the HA size. Data analysis revealed that TMD deletions alter the activity and polymer size. Following the deletion of all TMDs, the polysaccharide synthesis capability of the shortest form of the enzyme (HASIntra) was significantly preserved. The highest level of activity among the variants was for HAS_123_ (91% of WT). The results indicated that TMD_4_, TMD_5_, and the extracellular C-terminus region have no deleterious effects on HA synthesis. The results confirm earlier findings by Baggenstoss et al*.* and Yang et al*.* that C-terminus mutants are able to synthesize HA (Baggenstoss et al. 2017; Yang et al. 2017). SeHAS truncation by Baggenstoss et al*.* revealed that the deletion of residues 398–417 caused an undetectable HA MW. It is presumed that this mutant is unstable (not expressed), indicating that the HAS C-terminal region is likely critical for enzyme stability (Baggenstoss et al. 2017). In previous studies, the transmembrane topology of SeHAS revealed that the C-terminal peptide of SeHAS from residues 402 to 417 is located inside the cell (Yang et al. 2017). In this study, the topological prediction showed that the C-terminus of GGS-HAS is an extracellular domain, in which deletion of this region may have a different role in the enzyme activity and control of MW of polymer compared to similar studies.

Deletion of first TMD in HAS_23_ reduces the enzyme activity to 66% of WT. The comparison of HAS_123_ and HAS_23_ productivity demonstrated that, in contrast to HAS_123_*,* deletion of TMD_1_ has a significant effect on the HAS activity. It should be notified that in our previous work, we prepared.

HAS_Intra_, HAS_123_, HAS_23_, HAS_3_, and HAS_2_ variants and preliminary screening of activity elucidated the importance role TMD_1_, TMD_2_, and TMD_3_ for full activity (Cohan et al. 2023). One significant difference in the current work is the use of different purification methods for the variants that leads to obtaining higher specific activity for the variants. We used a hybrid purification method for HAS_Intra_, in which the resin is washed by graduate reduction of urea. This procedure resulted in better folding of the variant and its increased activity (38% versus 11%). For membrane variants, we tested different nonionic detergents such as DDM, Triton X-100, and Tween 20 to evaluate their effects on protein solubilization and activity. As our data demonstrated, among detergents, DDM showed the best results (the highest solubilization and activity) for the variants. It must be notified that the type of detergent is very important for isolation of membrane proteins and should be tested for each protein (Orwick-Rydmark et al. 2016). For example, in a study conducted by Lee et al*.*, different detergents including DDM, Brij-35, Triton X-100, cholate, CHAPSO, Zwittergent 3–12, Deoxy BIG CHAP, and digitonin were used for purification of cadherin-11 and it was found that Triton X-100 and DDM were more efficient than the others (Lee et al. 2018). Similar to our previous work, HAS_Intra_ (the central domain of SeHAS) showed a reduction in activity by up to 38%. Although this region as a single functional glycosyltransferase domain is able to bind to the substrates and catalyze the polymerization of HA but requires TMD_1_, TMD_2_, and TMD_3_ for full activity. In general, the activity measurements indicated that these TMDs are essential for accelerating the synthesis of the polymer.

Despite our previous study that clarified the effect of TMDs on the SeHAS activity, however, the role of the N-terminal region on the control of product size was not investigated so far. Therefore, in the current study, the effect of such deletions on the polymer size was assessed. Our results illuminated that in comparison to SeHAS, which produced a polydisperse HMW polymer, HAS_123_ and HAS_23_ produced polydisperse LMW-HAs. Meanwhile, HAS_Intra_ produced a low-disperse LMW-HA. Preparation of smaller HA products (< 30 kDa) by the variants compared to SeHAS may be due to the removal of TMDs in the N- and C-terminal of the enzyme that probably alters the conformation of the enzyme. The HA Mw produced by HAS_123_ and HAS_23_ was almost identical with very similar polydispersity. This result is not unexpected, since HAS_123_ and HAS_23_ show variation at only a position, including TMD_1_. Maybe it can be concluded that the first TMD may be more involved in the synthesis rate than the size control.

Of the biological activities of HA, the most interesting one is the angiogenesis capability of LMW-HA in wound repair (cell migration assay) through stimulating EC proliferation (Mo et al. 2011). Therefore, to test the biological activity of produced polymers, a cell proliferation assay was utilized. We observed all produced LMW-HAs (HA_123_, HA_23_, and HA_Intra_), as well as the control (HA_10kDa_) could stimulate EC proliferation in a dose-dependent manner with maximum value at 6 µg/mL after 72 h. The data also elucidated that this stimulatory effect was highest for HA_Intra_ and HA_10kDa_, which are low-disperse. This phenomenon highlights the importance of LMW low-disperse preparations of this polymer for medicinal applications. On the contrary, HA_SeHAS_ and HA_760kDa_ (control) showed only an inhibitory effect at 150 µg/mL without any stimulatory effect at lower concentrations. Previous studies evaluated the regulatory effect of HAs on HUVEC proliferation and indicated that ECs respond poorly to large HAs with a reduction in the growth at 100–500 µg/ml. While LMW-HAs have stimulatory effects on these cells at 3–20 μg/mL by interacting with receptor for HA-mediated motility (RHAMM) and CD44 as receptors (Ibrahim and Ramamurthi 2008; Mo et al. 2011; Queisser et al. 2021). In addition to the proliferation assay, we investigated the biological activity of polymers by a cell migration assay in vitro model (Gao et al. 2008). Interestingly, LMW-HAs were fully active for cell proliferation and migration. The cell migration test demonstrated that HA_123_, HA_23_, HA_Intra_, and HA_10kDa_ fully recover the lesion after 24 h at the lower dose (10 µg/mL). Nonetheless, in the case of HA_SeHAS_ and HA_760kDa_, the scratched area did not fully close. Cell imaging also demonstrated that an increase in the HA concentration postponed the healing of HUVEC, as all groups, independent in size, did not show a full recovery within 24 h. These results are in agreement with the literature data that LMW-HAs are strong inducers of angiogenesis and recover the EC wound with different origins at low concentrations (Gao et al. 2006, 2008; Mo et al. 2011; Slevin et al. 2002; Wang et al. 2016). Our data also confirms the opposing effects of LMW-HA at low (stimulatory) and high concentrations (inhibitory) in wound recovery (Gao et al. 2008). Our data suggested that the prepared LMW-HAs by the variants are beneficial for accelerating angiogenesis and wound repair.

This achievement should be highlighted for HAS_Intra_ because the expression and purification of membrane variants of the enzyme are very time and cost-consuming. Furthermore, non-membrane forms are very interesting for scale-up from an industrial point of view. On the other hand, a complete molecular-level understanding of biopolymer synthesis by SeHASs and the development of modification technologies will enable structure-guided enzyme engineering to synthesize HA polymers with defined MWs for wider application. The findings could also be extended to other glycosyltransferases with little functional and structural information.

### Supplementary Information

**Additional file 1**.

## Data Availability

Data are available upon reasonable request.

## Abbreviations

BCA: Bicinchoninic acid
BSA: Bovine serum albumin
DDM: N-dodecyl β-d-maltoside
ECs: Endothelial cells
FTIR: Fourier-transform infrared spectroscopy
GGS-S88: *Streptococcus* *equisimilis* Isolate S88 group G
GlcUA: Glucuronic acid
GlcNAc: N-acetyl glucoseamine
h: Hour
HA: Hyaluronic acid
HAS: Hyaluronan synthase enzyme
HMW-HA: High-molecular-weight HA
HUVEC: Umbilical vein endothelial cells
IPTG: Isopropyl β-d-1-thiogalactopyranoside
kDa: Kilo Dalton
LMW-HA: Low-molecular-weight HA
MDs: Membrane domains
MTT: Methylthiazolyldiphenyl-tetrazolium bromide
Mw: Molecular weight
PAGE: Polyacrylamide gel electrophoresis
PBS: Phosphate-buffered saline
PDI: Polydispersity index
SeHAS: *Streptococcus equisimilis* hyaluronan synthase enzyme
*S. equisimilis*: *Streptococcus equisimilis*
SDS: Sodium dodecyl sulfate
TMD: Trans membrane domain
UDP: Uridine diphosphate

## References

[CR1] Al-Khateeb R, Prpic J (2019). Hyaluronic acid: the reason for its variety of physiological and biochemical functional properties. Appl Clin Res Clin Trials Regul Affairs.

[CR2] Agarwal G, Kv K, Prasad SB, Bhaduri A, Jayaraman G (2019). Biosynthesis of hyaluronic acid polymer: dissecting the role of sub structural elements of hyaluronan synthase. Sci Rep.

[CR3] Amjad Zanjani FS, Afrasiabi S, Norouzian D, Ahmadian G, Hosseinzadeh SA, Fayazi Barjin A, Cohan RA, Keramati M (2022). Hyaluronic acid production and characterization by novel *Bacillus subtilis* harboring truncated Hyaluronan Synthase. AMB Express.

[CR4] Afrasiabi S, Zanjani FSA, Ahmadian G, Cohan RA, Keramati M (2023). The effect of manipulating glucuronic acid biosynthetic pathway in *Bacillus**subtilis* strain on hyaluronic acid production. AMB Express.

[CR5] Bitter T (1962). A modified uronic acid carbazole reaction. Anal Biochem.

[CR6] Boeriu CG, Springer J, Kooy FK, van den Broek LA, Eggink G (2013). Production methods for hyaluronan. Int J Carbohydr Chem.

[CR7] Baggenstoss BA, Harris EN, Washburn JL, Medina AP, Nguyen L, Weigel PH (2017). Hyaluronan synthase control of synthesis rate and hyaluronan product size are independent functions differentially affected by mutations in a conserved tandem B-X7-B motif. Glycobiology.

[CR8] Cowman MK (2017). Hyaluronan and hyaluronan fragments. Adv Carbohydr Chem Biochem.

[CR9] Chen H, Qin J, Hu Y (2019). Efficient degradation of high-molecular-weight hyaluronic acid by a combination of ultrasound, hydrogen peroxide, and copper ion. Molecules.

[CR10] Cavalcanti AD, Melo BA, Oliveira RC, Santana MH (2019). Recovery and purity of high molar mass bio-hyaluronic acid via precipitation strategies modulated by pH and sodium chloride. Appl Biochem Biotechnol.

[CR11] Chahuki FF, Aminzadeh S, Jafarian V, Tabandeh F, Khodabandeh M (2019). Hyaluronic acid production enhancement via genetically modification and culture medium optimization in *Lactobacillus acidophilus*. Int J Biol Macromol.

[CR12] Ciccone V, Zazzetta M, Morbidelli L (2019). Comparison of the effect of two hyaluronic acid preparations on fibroblast and endothelial cell functions related to angiogenesis. Cells.

[CR13] Cesaretti M, Luppi E, Maccari F, Volpi N (2003). A 96-well assay for uronic acid carbazole reaction. Carbohydr Polym.

[CR14] Cohan RA, Keramati M, Afshari E, Parsian P, Ahani R, Ebrahimi T (2023). Evaluation of transmembrane domain deletions on hyaluronic acid polymerization of hyaluronan synthase isolated from *Streptococcus equisimilis* group G. World J Microbiol Biotechnol.

[CR15] David-Raoudi M, Tranchepain F, Deschrevel B, Vincent JC, Bogdanowicz P, Boumediene K, Pujol JP (2008). Differential effects of hyaluronan and its fragments on fibroblasts: relation to wound healing. Wound Repair Regens.

[CR16] Deed R, Rooney P, Kumar P, Norton JD, Smith J, Freemont AJ, Kumar S (1997). Early-response gene signalling is induced by angiogenic oligosaccharides of hyaluronan in endothelial cells Inhibition by non-angiogenic, high-molecular-weight hyaluronan. Int J Cancer.

[CR17] Dodero A, Williams R, Gagliardi S, Vicini S, Alloisio M, Castellano M Characterization of hyaluronic acid by dynamic light scattering and rheological techniques. In: AIP conference proceedings, 2018. vol 1981. AIP Publishing LLC, p 020184

[CR18] Dovedytis M, Liu ZJ, Bartlett S (2020). Hyaluronic acid and its biomedical applications: a review. Eng Regen.

[CR19] Ebrahimi T, Keramati M, Ahangari Cohan R (2022). The strengths and weaknesses of methods for determination of hyaluronan molecular weight. Open Access J Microbiol Biotechnol.

[CR20] Garantziotis S, Savani RC (2019). Hyaluronan biology: a complex balancing act of structure, function, location and context. Matrix Biol.

[CR21] Gilli R, Kacuráková M, Mathlouthi M, Navarini L, Paoletti S (1994). FTIR studies of sodium hyaluronate and its oligomers in the amorphous solid phase and in aqueous solution. Carbohydr Res.

[CR22] Gao F, Cao M, Yang C, He Y, Liu Y (2006). Preparation and characterization of hyaluronan oligosaccharides for angiogenesis study. J Biomed Mater Res Part B.

[CR23] Gao F, Yang C, Mo W, Liu Y, He Y (2008). Hyaluronan oligosaccharides are potential stimulators to angiogenesis via RHAMM mediated signal pathway in wound healing. Clin Investig Med.

[CR24] Hill AV (1910). A new mathematical treatment of changes of ionic concentration in muscle and nerve under the action of electric currents, with a theory as to their mode of excitation. J Physiol.

[CR25] Heldermon C, DeAngelis PL, Weigel PH (2001). Topological organization of the hyaluronan synthase from *streptococcus pyogenes*. J Biol Chem.

[CR26] Hascall VC, Wang A, Tammi M, Oikari S, Tammi R, Passi A, Vigetti D, Hanson RW, Hart GW (2014). The dynamic metabolism of hyaluronan regulates the cytosolic concentration of UDP-GlcNAc. Matrix Biol.

[CR27] Hofmann K (1993). TMbase-A database of membrane spanning proteins segments. Biol Chem Hoppe-Seyler.

[CR28] Ibrahim S, Ramamurthi A (2008). Hyaluronic acid cues for functional endothelialization of vascular constructs. J Tissue Eng Regen Med.

[CR29] Ikegami-Kawai M, Takahashi T (2002). Microanalysis of hyaluronan oligosaccharides by polyacrylamide gel electrophoresis and its application to assay of hyaluronidase activity. Anal Biochem.

[CR30] Jokela TA, Makkonen KM, Oikari S, Kärnä R, Koli E, Hart GW, Tammi RH, Carlberg C, Tammi MI (2011). Cellular content of UDP-N-acetylhexosamines controls hyaluronan synthase 2 expression and correlates with O-linked N-acetylglucosamine modification of transcription factors YY1 and SP1. J Biol Chem.

[CR31] Jafari B, Keramati M, Ahangari Cohan R, Atyabi SM, Ali Hosseinzadeh S (2022). Development of *Streptococcus equisimilis* Group G mutant strains with ability to produce low polydisperse and low-molecular-weight hyaluronic acid. Iran Biomed J.

[CR32] Kumari K, Weigel PH (2005). Identification of a membrane-localized cysteine cluster near the substrate-binding sites of the *Streptococcus equisimilis* hyaluronan synthase. Glycobiology.

[CR33] Kumar S, West DC, Ponting JM, Gattamaneni H (1989). Sera of children with renal tumours contain low-molecular-mass hyaluronic acid. Int J Cancer.

[CR34] Kooy FK, Beeftink HH, Eppink MH, Tramper J, Eggink G, Boeriu CG (2014). Kinetic and structural analysis of two transferase domains in *Pasteurella multocida* hyaluronan synthase. J Mol Catal B Enzym.

[CR35] Karami M, Shahraky MK, Ranjbar M, Tabandeh F, Morshedi D, Aminzade S (2021). Preparation, purification, and characterization of low-molecular-weight hyaluronic acid. Biotech Lett.

[CR36] Keramati M, Cohan RA, Parsian P, Afshari E (2022) Truncated hyaluronan synthase and polynucleotide encoding the same. Google Patents

[CR37] Kumari K, Tlapak-Simmons VL, Baggenstoss BA, Weigel PH (2002). The streptococcal hyaluronan synthases are inhibited by sulfhydryl-modifying reagents, but conserved cysteine residues are not essential for enzyme function. J Biol Chem.

[CR38] Kumari K, Baggenstoss BA, Parker AL, Weigel PH (2006). Mutation of two intramembrane polar residues conserved within the hyaluronan synthase family alters hyaluronan product size. J Biol Chem.

[CR39] Liu L, Liu Y, Li J, Du G, Chen J (2011). Microbial production of hyaluronic acid: current state, challenges, and perspectives. Microb Cell Fact.

[CR40] Lee Y-C, Bååth JA, Bastle RM, Bhattacharjee S, Cantoria MJ, Dornan M, Gamero-Estevez E, Ford L, Halova L, Kernan J (2018). Impact of detergents on membrane protein complex isolation. J Proteome Res.

[CR41] Min H, Cowman MK (1986). Combined alcian blue and silver staining of glycosaminoglycans in polyacrylamide gels: application to electrophoretic analysis of molecular weight distribution. Anal Biochem.

[CR42] Markovitz A, Cifonelli J, Dorfman A (1959). The biosynthesis of hyaluronic acid by group A *Streptococcus*: VI. Biosynthesis from uridine nucleotides in cell-free extracts. J Biol Chem.

[CR43] Merril CR, Goldman D, Sedman SA, Ebert MH (1981). Ultrasensitive stain for proteins in polyacrylamide gels shows regional variation in cerebrospinal fluid proteins. Science.

[CR44] Mo W, Yang C, Liu Y, He Y, Wang Y, Gao F (2011). The influence of hyaluronic acid on vascular endothelial cell proliferation and the relationship with ezrin/merlin expression. Acta Biochim Biophys Sin.

[CR45] Moretto P, Karousou E, Viola M, Caon I, D’Angelo ML, De Luca G, Passi A, Vigetti D (2015). Regulation of hyaluronan synthesis in vascular diseases and diabetes. J Diabetes Res.

[CR46] Mandawe J, Schwaneberg U, Blank LM (2018) Engineering of hyaluronic acid synthases from *Streptococcus equi* subsp. zooepidemicus and *Pasteurella multocida* towards improved HA chain length and titer. Universitätsbibliothek der RWTH Aachen

[CR47] Orwick-Rydmark M, Arnold T, Linke D (2016). The use of detergents to purify membrane proteins. Curr Protoc Protein Sci.

[CR48] Prosdocimi M, Bevilacqua C (2012). Exogenous hyaluronic acid and wound healing: an updated vision. Panminerva Med.

[CR49] Queisser KA, Mellema RA, Petrey AC (2021). Hyaluronan and its receptors as regulatory molecules of the endothelial interface. J Histochem Cytochem.

[CR50] Rodriguez-Marquez CD, Arteaga-Marin S, Rivas-Sánchez A, Autrique-Hernández R, Castro-Muñoz R (2022). A review on current strategies for extraction and purification of hyaluronic acid. Int J Mol Sci.

[CR51] Stoolmiller A, Dorfman A (1969). The biosynthesis of hyaluronic acid by *Streptococcus*. J Biol Chem.

[CR52] Sambrook J, Fritsch EF, Maniatis T (1989) Molecular cloning: a laboratory manual. Cold spring harbor laboratory press

[CR53] Smith P, Krohn RI, Hermanson G, Mallia A, Gartner F, Provenzano M, Fujimoto E, Goeke N, Olson B, Klenk D (1985). Measurement of protein using bicinchoninic acid. Anal Biochem.

[CR54] Sattar A, Rooney P, Kumar S, Pye D, West DC, Scott I, Ledger P (1994). Application of angiogenic oligosaccharides of hyaluronan increases blood vessel numbers in rat skin. J Investig Dermatol.

[CR55] Slevin M, Kumar S, Gaffney J (2002). Angiogenic oligosaccharides of hyaluronan induce multiple signaling pathways affecting vascular endothelial cell mitogenic and wound healing responses. J Biol Chem.

[CR56] Sousa AS, Guimarães AP, Gonçalves CV, Silva IJ, Cavalcante CL, Azevedo DC (2009). Purification and characterization of microbial hyaluronic acid by solvent precipitation and size-exclusion chromatography. Sep Sci Technol.

[CR57] Simpson MA, De La Motte C, Sherman LS, Weigel PH (2015) Advances in hyaluronan biology: signaling, regulation, and disease mechanisms. vol 2015. Hindawi 10.1155/2015/690572 PMC4584047 26446415

[CR58] Schulte S, Doss SS, Jeeva P, Ananth M, Blank LM, Jayaraman G (2019). Exploiting the diversity of streptococcal hyaluronan synthases for the production of molecular weight–tailored hyaluronan. Appl Microbiol Biotechnol.

[CR59] Song C, You Y, Wen C, Fu Y, Yang J, Zhao J, Song S (2023). Characterization and gel properties of low-molecular-weight carrageenans prepared by photocatalytic degradation. Polymers.

[CR60] Suarez-Arnedo A, Figueroa FT, Clavijo C, Arbeláez P, Cruz JC, Muñoz-Camargo C (2020). An image J plugin for the high throughput image analysis of in vitro scratch wound healing assays. PLoS ONE.

[CR61] Tavianatou AG, Caon I, Franchi M, Piperigkou Z, Galesso D, Karamanos NK (2019). Hyaluronan: molecular size-dependent signaling and biological functions in inflammation and cancer. FEBS J.

[CR62] Tlapak-Simmons VL, Baggenstoss BA, Clyne T, Weigel PH (1999). Purification and lipid dependence of the recombinant hyaluronan synthases from *streptococcus pyogenes* and *streptococcus equisimilis*. J Biol Chem.

[CR63] Tlapak-Simmons VL, Baggenstoss BA, Kumari K, Heldermon C, Weigel PH (1999). Kinetic characterization of the recombinant hyaluronan synthases from *Streptococcus pyogenes* and *Streptococcus equisimilis*. J Biol Chem.

[CR64] Tlapak-Simmons VL, Baron CA, Weigel PH (2004). Characterization of the purified hyaluronan synthase from *Streptococcus equisimilis*. Biochemistry.

[CR65] West D, Kumar S (1988). Endothelial cell proliferation and diabetic retinopathy. The Lancet.

[CR66] West DC, Kumar S (1989). The effect of hyaluronate and its oligosaccharides on endothelial cell proliferation and monolayer integrity. Exp Cell Res.

[CR67] West DC, Kumar S (1991). Tumour-associated hyaluronan: a potential regulator of tumour angiogenesis. Int J Radiat Biol.

[CR68] Weigel PH, Baggenstoss BA (2012). Hyaluronan synthase polymerizing activity and control of product size are discrete enzyme functions that can be uncoupled by mutagenesis of conserved cysteines. Glycobiology.

[CR69] West DC, Hampson IN, Arnold F, Kumar S (1985). Angiogenesis induced by degradation products of hyaluronic acid. Science.

[CR70] Wang Y, Han G, Guo B, Huang J (2016). Hyaluronan oligosaccharides promote diabetic wound healing by increasing angiogenesis. Pharmacol Rep.

[CR71] Xu X-M, Chen Y, Chen J, Yang S, Gao F, Underhill CB, Creswell K, Zhang L (2003). A peptide with three hyaluronan binding motifs inhibits tumor growth and induces apoptosis. Cancer Res.

[CR72] Yang J, Cheng F, Yu H, Wang J, Guo Z, Stephanopoulos G (2017). Key role of the carboxyl terminus of hyaluronan synthase in processive synthesis and size control of hyaluronic acid polymers. Biomacromol.

[CR73] Yao Z-Y, Qin J, Gong J-S, Ye Y-H, Qian J-Y, Li H, Xu Z-H, Shi J-S (2021). Versatile strategies for bioproduction of hyaluronic acid driven by synthetic biology. Carbohydr Polym.

