# Supplementary material for: Enzyme variants in biosynthesis and biological assessment of different molecular weight hyaluronan
Source: AMB Express. 2024 May 10;14:56. doi: 10.1186/s13568-024-01713-4 (PMC11087452; doi:10.1186/s13568-024-01713-4)
Supplement: Supplementary file 1 — Additional file 1. [file 13568_2024_1713_MOESM1_ESM.docx]

**Supplementary data**

**Table S1.**  Primers used for amplifying SeHAS and the variants in this study.

| **Primer Sequence**  **5' 3'** | **Deleted domains** | **Construct name** |
| --- | --- | --- |
| **F**: ggaattc**CATATG**agaacattaaaaaacctcataactg  **R**: cccg**CTCGAG**taataattttttacgtgttcc | None | SeHAS |
| **F**: acg**CATATG**agaacattaaaaaacctc  **R**: cga**CTCGAG**atcaaattctctgacattgc | TMD5 TMD4 | HAS_123_ |
| **F**: gat**CATATG**gctaaaggaagcttgtcaatttatgg  **R**: cga**CTCGAG**atcaaattctctgacattgc | TMD1  TMD4 TMD5 | HAS_23_ |
| **F**: tac**CATATG**aagccatttaagggaagg  **R**: ata**CTCGAG**aggattgttcatgattttcttaacag | TMD1 TMD2  TMD3  TMD4  TMD5 | HAS_Intra_ |

**Table S2.** The characteristics of SeHAS and variants.

| **Enzyme** | **Transmembrane membrane domain** | **Start** | **End** | **Length (AA)** | **MW (kDa)** |
| --- | --- | --- | --- | --- | --- |
| SeHAS | 1, 2, 3, 4, 5 | 1 | 417 | 417 | 42.0 |
| HAS_123_ | 1, 2, 3 | 1 | 341 | 341 | 39.1 |
| HAS_23_ | 2, 3 | 33 | 341 | 308 | 35.9 |
| HAS_Intra_ | None | 55 | 319 | 264 | 30.0 |

**Table S3.** Solubilization and activity (HA titer) of variants after purification by different detergents.

| **HA Titer (µg/mL)** | | | **Protein Concentration (µg/mL)** | | | **Detergent** |
| --- | --- | --- | --- | --- | --- | --- |
| HAS_23_ | HAS_123_ | SeHAS | HAS_23_ | HAS_123_ | SeHAS |  |
| 104.70 ± 3.4 | 160.09 ± 0.8 | 225.24 ± 1.4 | 997.96 ± 5.7 | 886.11 ± 1.7 | 1378.70 ± 8.2 | DDM |
| 12.25 ± 4.1 | 18.36 ± 3.6 | 67.22 ± 8.7 | 301.45 ± 1.6 | 206.44 ± 2.5 | 774.86 ± 4.4 | Triton X-100 |
| 6.05 ± 2.3 | 10.75 ± 2.0 | 56.32 ± 8.1 | 108.22 ± 2.3 | 150.62 ± 1.2 | 644.64 ± 3.4 | Tween 20 |


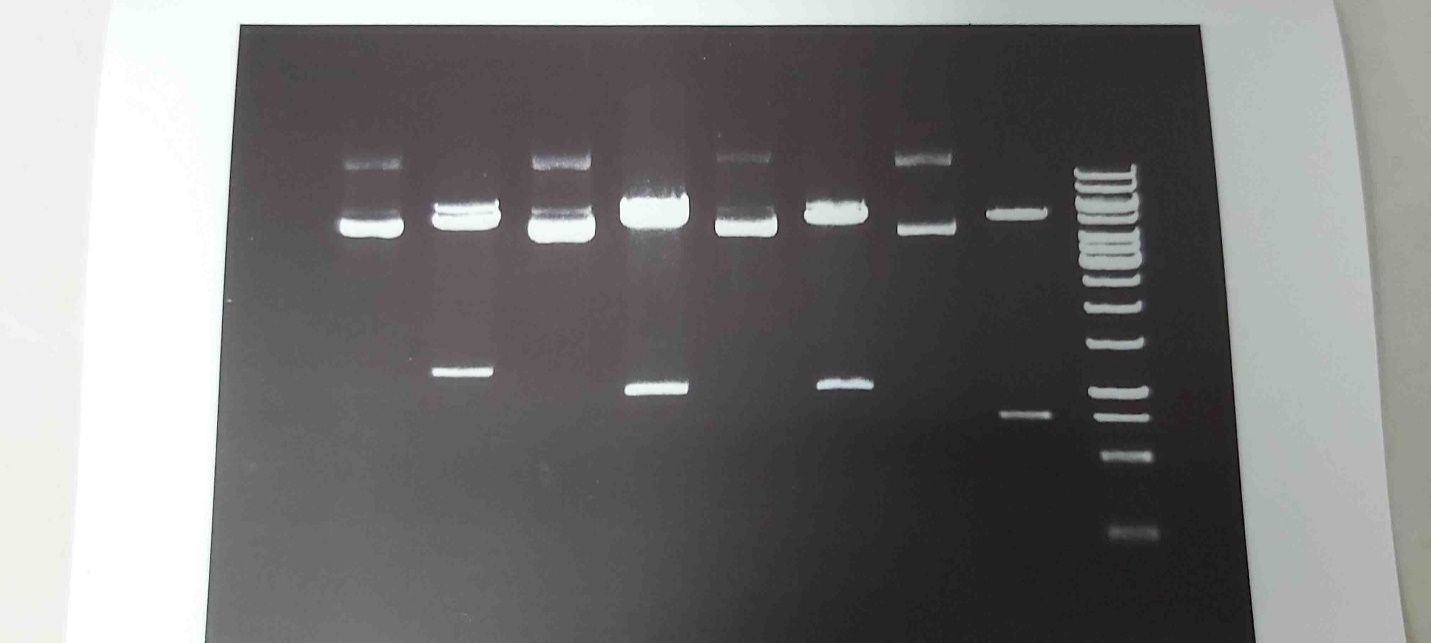

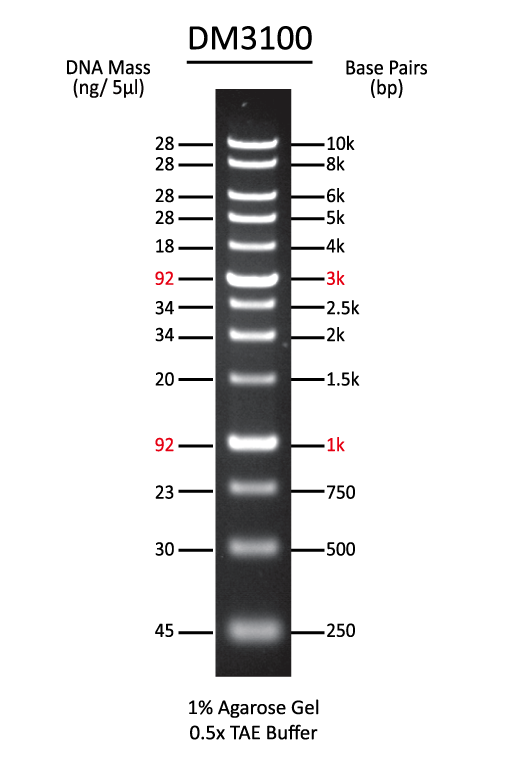


1271 bp

807 bp

1065 bp

981 bp

1 2 3 4 5 6 7 8 M

**Fig. S1.** Restriction map analysis of recombinant plasmids using *Xba*I and *Nde*I restriction enzymes [Lane 1: undigested pET-28a(+)-SeHAS plasmid, Lane 2: digested pET-28a(+)-SeHAS, Lane 3: undigested pET-28a(+)-HAS_23_ plasmid, Lane 4: digested pET-28a(+)-HAS_23_ plasmid, Lane 5: undigested pET-28a(+)-HAS_123_ plasmid, Lane 6: digested pET-28a(+)-HAS_123_ plasmid, Lane 7: undigested pET-28a(+)-HAS_Intra_ plasmid, Lane 8: digested pET-28a(+)-HAS_Intra_ plasmid, and M: DNA marker.]


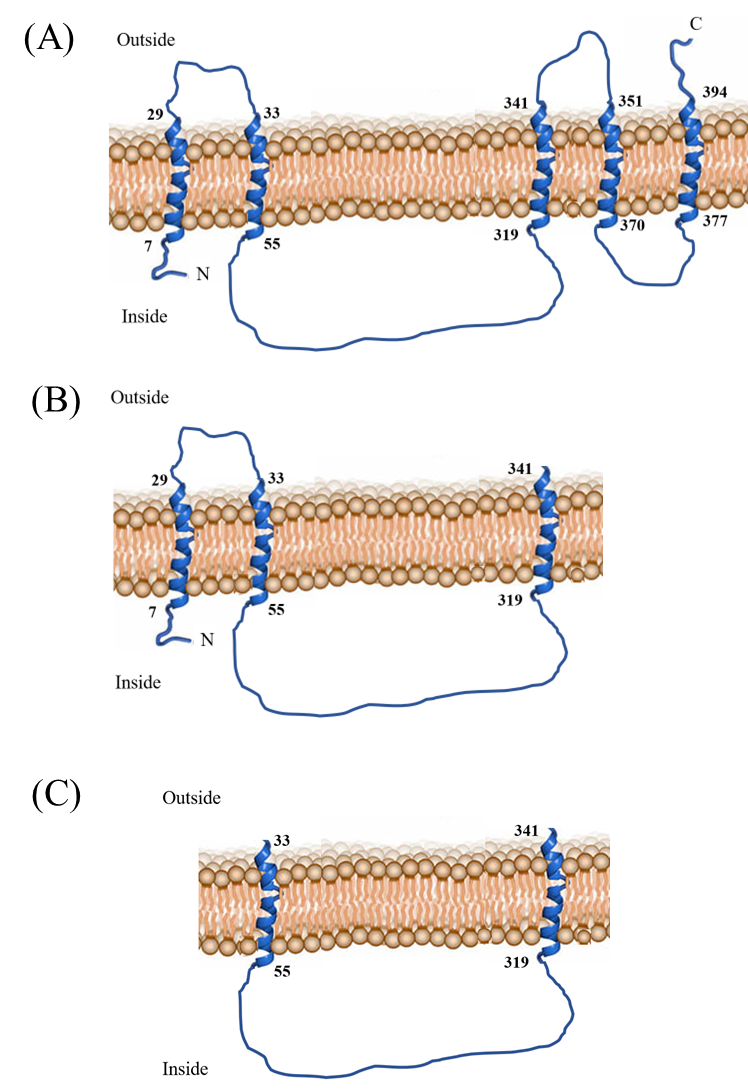


**Fig. S2.** Membrane-topology models for (**A**) seHAS, (**B**) HAS_123_, and (**C**) HAS_12_. The numbers within each TMD indicate the starting and ending residues.


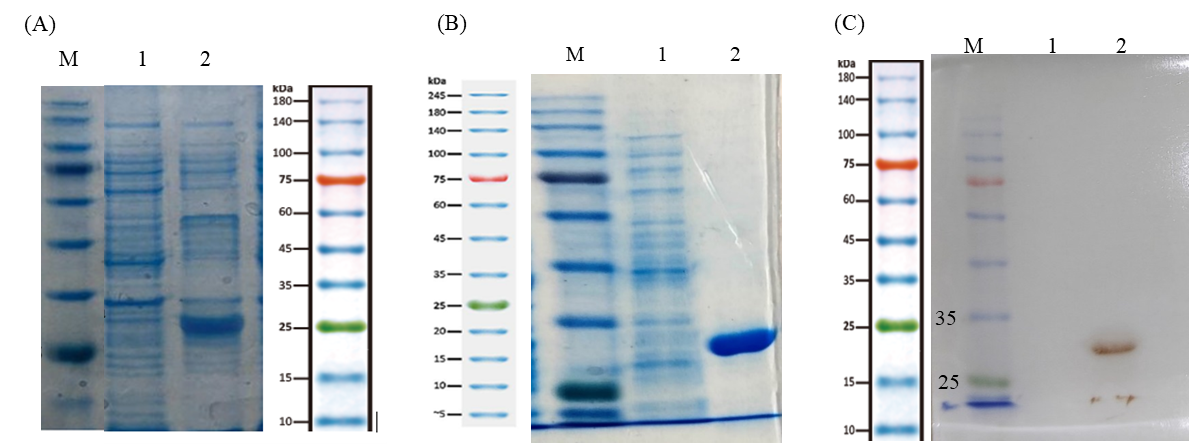


# Fig. S3. 12% SDS-PAGE and western blot analysis of recombinant HAS_Intra_. [(A) Lane 1: uninduced cell lysate; Lane 2: induced cell lysate. (B) Lane 1: uninduced cell lysate; Lane 2: eluted fraction, (C) Lane 1: uninduced cell lysate; Lane2: eluted fraction, and Lane M; protein marker].

**
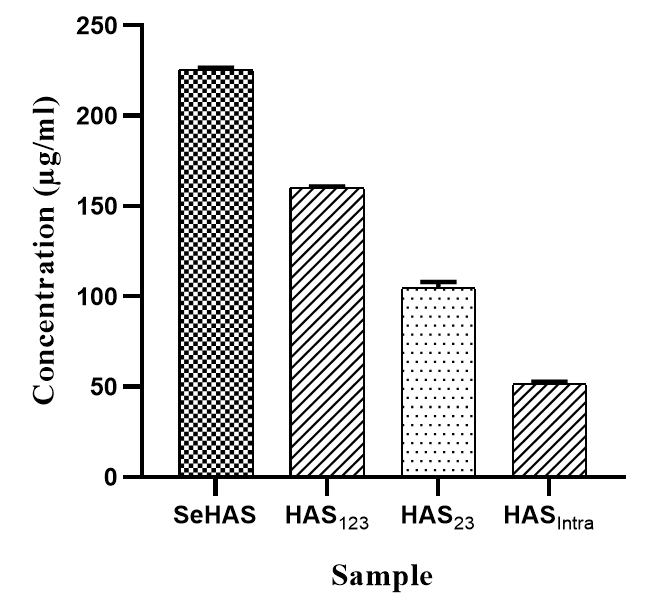
**

**Fig. S4.** Quantitative measurement of HA synthesized in the enzymatic reactions after purification. Data are represented as Mean ± SD from three independent measurements.


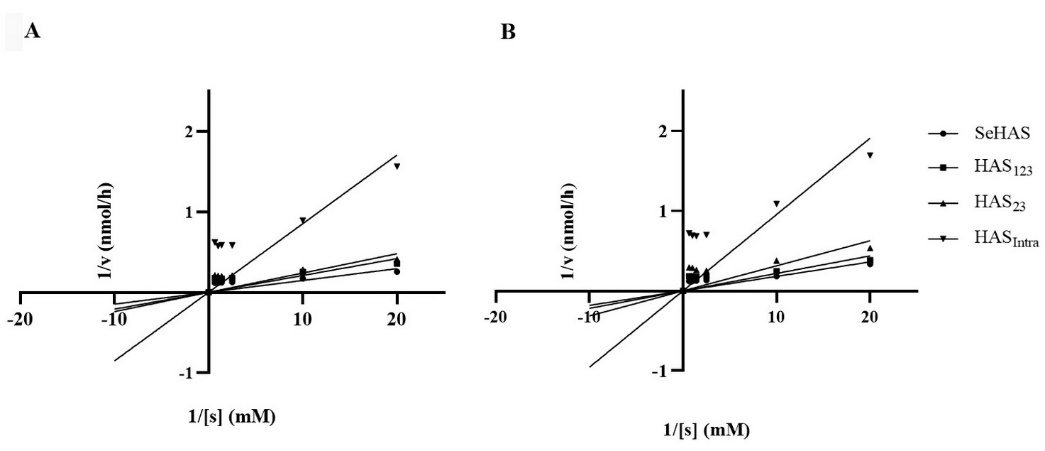


**Fig. S5.** The Lineweaver-Burk plots **show the** effect of substrate concentration on the activity of SeHAS variants at various concentrations of (A) UDP-GlcUA and (B) UDP-GLcNAc. The Vmax values were determined by varying the concentration of one substrate from 0.05 to 1.5 mM while keeping the other at 1.5 mM.


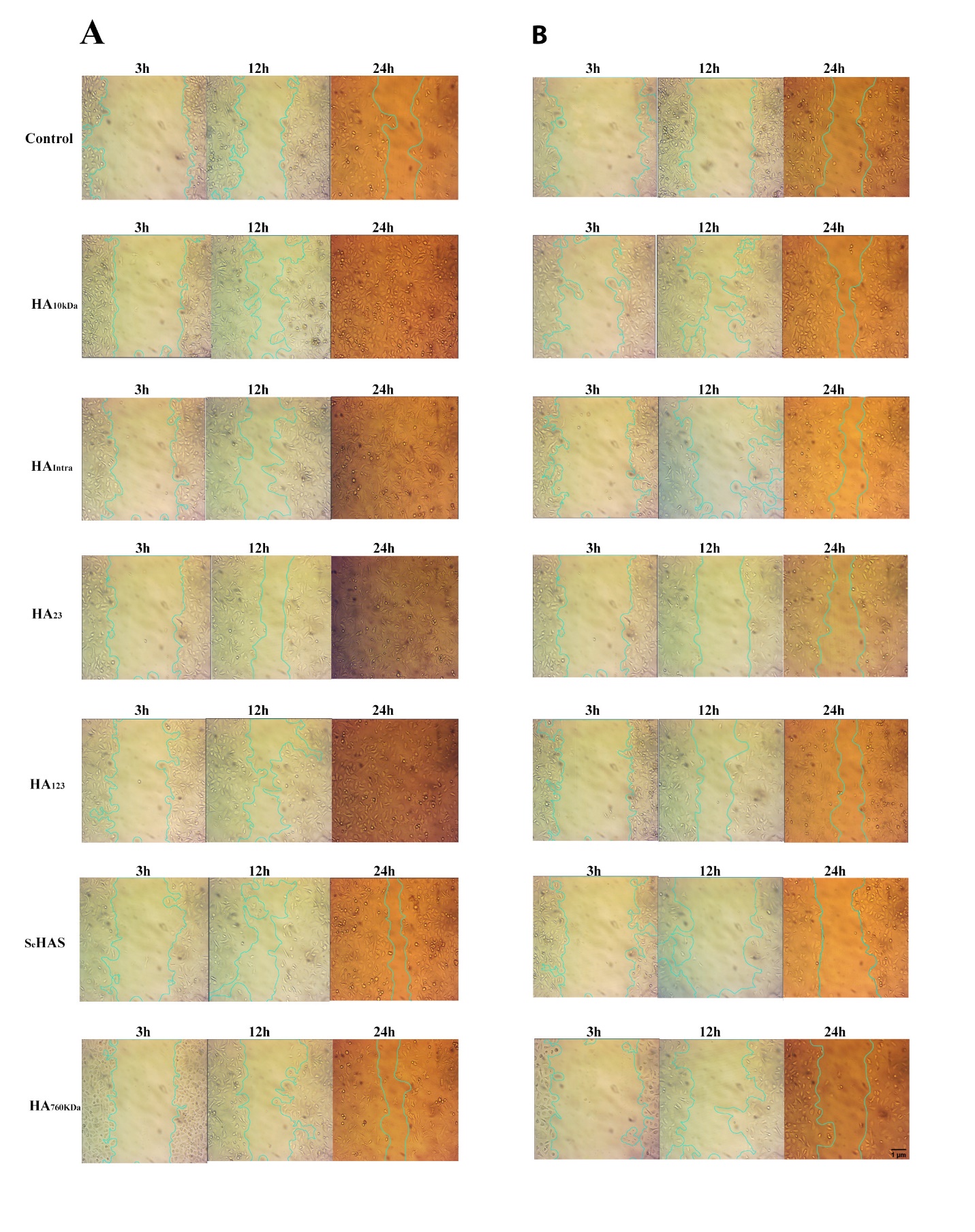


**Fig. S6**. Cell imaging of cell migration assay. The images show the influence of LMW-HAs and HMW-HAs at a concentration of 10 μg/mL (A) and 200 μg/mL (B) on ECs proliferation to recover the lesion after 3, 12, and 24 h in comparison to untreated control (518*545 pixels).
